# The triglyceride-high-density lipoprotein-glucose-body index: a superior novel biomarker for diabetic kidney disease in type 2 diabetes

**DOI:** 10.3389/fendo.2025.1749826

**Published:** 2026-01-12

**Authors:** Jian Yang, Bingsong Xie, Zhiling Deng, Zhifu Zhang, Hairong Zhou

**Affiliations:** 1 Department of General Medicine, Longhua District People’s Hospital, Shenzhen, China; 2 Department of General Medicine, The Eighth Affiliated Hospital of Sun Yat-sen University, Shenzhen, China

**Keywords:** associative, diabetic kidney disease, insulin resistance, TyHGB index, type 2 diabetes

## Abstract

**Background:**

The triglyceride-glucose (TyG) index is a recognized surrogate marker of insulin resistance but lacks integration of high-density lipoprotein cholesterol (HDL-C) and adiposity measures, which are pivotal in the pathogenesis of diabetic kidney disease (DKD). The novel triglyceride-high-density lipoprotein-glucose-body (TyHGB) index, combining TG/HDL-C ratio, fasting blood glucose (FBG), and body mass index (BMI), may offer a more comprehensive metabolic profile. This study aimed to evaluate the associative value of TyHGB for DKD in type 2 diabetes mellitus (T2DM) patients.

**Methods:**

A retrospective cross-sectional analysis of 1,382 adults with T2DM was conducted. We employed multivariable logistic regression, restricted cubic spline (RCS) analysis, and subgroup analyses to assess the independent and non-linear association of the TyHGB index with DKD. Receiver operating characteristic (ROC) curves, net reclassification improvement (NRI), and integrated discrimination improvement (IDI) were used to evaluate and compare its associative performance against the TyG index.

**Results:**

Among the participants, 286 (20.7%) were diagnosed with DKD. After full adjustment for demographic, clinical, and biochemical confounders, TyHGB was independently associated with DKD (OR = 1.11, 95%CI:1.05-1.17, p<0.001). RCS analysis revealed a significant non-linear relationship, with a sharp increase in DKD risk beyond a TyHGB threshold of 8.74. The TyHGB index demonstrated superior discriminative ability (AUC = 0.775, 95% CI: 0.747-0.803) compared to the TyG index (AUC = 0.644, p<0.001). Incorporating TyHGB into a baseline clinical model significantly improved risk association (AUC increased from 0.715 to 0.788, p<0.001) and provided substantial reclassification improvement (NRI = 0.647, IDI = 0.067).

**Conclusion:**

The TyHGB index exhibits a robust, independent, and non-linear association with DKD risk in T2DM patients and outperforms the established TyG index. As a readily accessible composite metric, it holds significant promise as a superior tool for early identification and risk stratification of DKD in clinical practice.

## 1 Introduction

The escalating global prevalence of Type 2 diabetes mellitus (T2DM) presents a paramount challenge to public health systems worldwide. According to International Diabetes Federation estimates, approximately 537 million adults were living with diabetes in 2021, with projections indicating this figure will rise to 783 million by 2045 (1). A particularly concerning complication is diabetic kidney disease (DKD), which affects approximately 40% of individuals with diabetes (2) and represents the principal cause of chronic kidney disease and end-stage renal disease globally (3). Beyond substantially elevating cardiovascular event risks and all-cause mortality (4, 5), DKD imposes devastating financial burdens on healthcare systems and society at large (6). Consequently, early identification of high-risk individuals and implementation of timely interventions hold critical importance for both clinical practice and public health initiatives.

DKD arises from multifactorial pathogenesis, characterized by a complex interplay among metabolic imbalances, hemodynamic changes, chronic inflammatory states, and oxidative stress (7, 8). Insulin resistance (IR) serves as a fundamental pathophysiological mechanism in both T2DM development and DKD progression, promoting renal injury through processes including glomerular hyperfiltration, endothelial dysfunction, and enhanced fibrogenesis (9). Although gold-standard IR assessment methods like the hyperinsulinemic-euglycemic clamp offer high accuracy (10), their technical complexity and substantial costs limit routine clinical application. This limitation has motivated the development of straightforward surrogate biomarkers derived from commonly measured clinical parameters.

Among emerging biomarkers, the triglyceride-glucose (TyG) index—calculated as Ln[fasting triglycerides (TG, mg/dL)×fasting blood glucose (FBG, mg/dL)/2]—has gained recognition as a reliable and readily accessible IR indicator (11). Numerous studies have documented strong associations between the TyG index and various metabolic disorders, including non-alcoholic fatty liver disease (NAFLD) and cardiovascular diseases (12, 13). Despite these advantages, the TyG index exhibits certain limitations. Its primary focus on glucose and triglyceride metabolism potentially overlooks other crucial pathophysiological pathways contributing to DKD. For instance, high-density lipoprotein cholesterol (HDL-C) demonstrates anti-inflammatory, antioxidant, and endothelial-protective properties, with low HDL-C levels independently correlating with albuminuria development and renal function decline (14, 15). Additionally, obesity—typically quantified by body mass index (BMI)—represents a well-established DKD risk factor that promotes renal damage through mechanisms such as hyperfiltration, leptin resistance, and chronic inflammation (16). The conventional TyG index fails to incorporate these two critical dimensions of HDL-C metabolism and adiposity, thereby constraining its comprehensive risk stratification potential.

To address these limitations, researchers recently proposed a novel composite index: the triglyceride high-density cholesterol-glucose body index (TyHGB). The TyHGB index is defined by the formula: TG/HDL-C + 0.7×FBG (mmol/L) + 0.1×BMI (kg/m²). This formula, including the specific coefficients (0.7 and 0.1) for FBG and BMI, was originally developed and validated by Xu et al. (17) through multivariate linear regression analysis, aiming to optimize the assessment of insulin resistance in the context of gestational diabetes mellitus. This single metric thereby encapsulates the key dimensions of dyslipidemia, dysglycemia, and adiposity. Initially validated for assessing insulin resistance in gestational diabetes mellitus, TyHGB has subsequently demonstrated superior performance over the TyG index in associating with NAFLD across diverse populations (18, 19). Since all required components are routinely measured in standard clinical practice, TyHGB represents a cost-effective and readily implementable tool.

Given the shared metabolic underpinnings of NAFLD and DKD—particularly regarding IR, dyslipidemia, and chronic inflammation—we hypothesize that TyHGB may similarly serve as a powerful marker for DKD in T2DM patients. However, no investigation to date has examined the relationship between TyHGB and DKD. Therefore, this study aimed to comprehensively evaluate TyHGB’s associative value for DKD in a large T2DM patient cohort, while comparing its performance against the established TyG index. To date, no established clinical cut-off points for TyHGB exist, and its association with DKD remains unexplored.

## 2 Materials and methods

### 2.1 Study design and population

We conducted a retrospective cross-sectional analysis using de-identified electronic health records sourced from a network of community health centers. The investigation included 1,382 adults diagnosed with type 2 diabetes mellitus who received medical services between 2023 and 2024. Data collection encompassed five community health centers affiliated with the Longhua District People’s Hospital of Shenzhen and one center under the Eighth Affiliated Hospital of Sun Yat-sen University (Futian).

Eligibility criteria required participants to be: (1) aged 18 years or older, and (2) diagnosed with T2DM according to American Diabetes Association standards (20). Exclusion criteria encompassed: (1) diagnosis of type 1 diabetes, gestational diabetes, or other specific diabetes types; (2) pre-existing chronic kidney disease from non-diabetic etiologies (e.g., glomerulonephritis, polycystic kidney disease); (3) end-stage renal disease requiring renal replacement therapy; (4) acute diabetic complications (including ketoacidosis or hyperosmolar state) at data collection; (5) severe systemic conditions such as heart failure (NYHA Class III/IV), advanced liver cirrhosis, autoimmune diseases, hematological malignancies, or active cancer; (6) active infectious diseases, systemic inflammatory conditions, or those on immunosuppressants; and (7) incomplete data necessary for TyHGB index calculation or DKD diagnosis.

### 2.2 Ethics statement

The study protocol obtained approval from the Medical Ethics Committee of Longhua District People’s Hospital of Shenzhen (Approval No.: Longhua District People’s Hospital (No [2025].087)). Formal data access authorization was secured through a written agreement with the Data Governance Committee of the Eighth Affiliated Hospital of Sun Yat-sen University (Futian) (Approval No.:SYSU8H-DGC- [2025]DA-013). All data handling complied strictly with institutional security protocols. All data handling complied with institutional security protocols and relevant data protection regulations, including the Protection of Personal Information Act (POPIA) where applicable. The research adhered fully to ethical principles outlined in the Declaration of Helsinki and relevant international guidelines governing human subjects research and retrospective data analysis. The ethics committees waived individual informed consent requirements due to the use of de-identified retrospective data.

It is hereby clarified that this ethical approval (2025) pertains specifically to the retrospective analysis of pre-existing data from the 2023–2024 period.

### 2.3 Data collection and variable definitions

The electronic health records provided information across three domains:

Demographics and History: Sex, age, T2DM duration, hypertension history, cardiovascular disease, stroke, smoking status, and alcohol consumption.

Anthropometric Measures: Body mass index (BMI), computed as weight in kilograms divided by height in meters squared (kg/m²).

Laboratory Parameters: Fasting blood glucose (FBG), glycated hemoglobin (HbA1c), total bilirubin (TBIL), alanine aminotransferase (ALT), aspartate aminotransferase (AST), triglycerides (TG), total cholesterol (TC), high-density lipoprotein cholesterol (HDL-C), low-density lipoprotein cholesterol (LDL-C), blood urea nitrogen (BUN), serum creatinine (SCr), uric acid (UA), white blood cell count (WBC), and platelet count (PLT).

We calculated the novel metabolic index, the triglyceride high-density cholesterol-glucose body index (TyHGB), according to the following formula: TyHGB = TG (mmol/L)/HDL-C (mmol/L) + 0.7×FBG (mmol/L) + 0.1×BMI (kg/m²). For comparative purposes, we computed the established Triglyceride-Glucose (TyG) index as: TyG = Ln [TG (mg/dL)×FBG (mg/dL)/2]. Note that the original formulas for TyHGB and TyG indices were developed using different units systems (mmol/L and mg/dL, respectively). In our calculations, all laboratory values were recorded in mmol/L. To compute the TyG index according to its standard formula, TG and FBG values were converted to mg/dL using the standard conversion factors (TG: 1 mmol/L = 88.57 mg/dL; FBG: 1 mmol/L = 18 mg/dL).

### 2.4 Outcome definition

The primary outcome was diabetic kidney disease (DKD) presence (21). The diagnosis required meeting one of two criteria: (1) a urinary albumin-to-creatinine ratio (UACR) of ≥30 mg/g, confirmed on at least two of three tests over a 3–6 month period without other identifiable causes; or (2) a persistently reduced estimated glomerular filtration rate (eGFR) below 60 mL/min/1.73 m² for over three months, as determined by the CKD-EPI equation. Based on these criteria, we categorized participants into DKD and non-DKD groups.

### 2.5 Statistical analysis

All statistical analyses were carried out with R software (version 4.3.1; R Foundation for Statistical Computing). A two-sided p-value < 0.05 indicated statistical significance. Normality of continuous variables was assessed using the Shapiro-Wilk test.

Baseline Characteristics: For continuous variables, normally distributed data are summarized as mean ± standard deviation, while non-normally distributed data are reported as median with interquartile range. Group comparisons were performed using the Student’s t-test and the Mann-Whitney U test, respectively. We expressed categorical variables as frequencies (percentages) and compared them via Chi-square tests.

Multivariable Logistic Regression: To quantify the independent association between TyHGB and DKD, we employed multivariable logistic regression with three sequentially adjusted models: Model 1 accounted for basic demographics (age and sex); Model 2 added lifestyle factors and comorbidities (hypertension, cardiovascular disease, stroke, smoking status, alcohol consumption, and BMI); Model 3 represented the primary fully adjusted model, which incorporated HbA1c in addition to Model 2 variables. Renal function markers (serum creatinine and blood urea nitrogen) were intentionally excluded from this primary model to avoid over-adjustment bias, as they are on the causal pathway of the outcome. To assess the robustness of our findings, we conducted a sensitivity analysis (Model 3a) that additionally adjusted for serum creatinine and blood urea nitrogen. Results appear as odds ratios (ORs) with corresponding 95% confidence intervals (CIs).

Restricted Cubic Spline (RCS) Analysis: We explored potential non-linear relationships between continuous TyHGB values and DKD risk by fitting multivariable logistic regression models incorporating RCS with four knots (at the 5th, 35th, 65th, and 95th percentiles). The likelihood ratio test assessed non-linearity significance.

Subgroup Analysis and Interaction: To evaluate association robustness across patient strata, we conducted subgroup analyses based on sex, BMI (<24 vs≥24 kg/m²), and hypertension status. After introducing interaction terms between TyHGB and subgroup variables into multivariable models, we used likelihood ratio tests to determine interaction significance.

Predictive Performance and Model Comparison: Using receiver operating characteristic (ROC) curve analysis, we evaluated and compared the associative abilities of TyHGB, the TyG index, and other individual indicators for DKD. We calculated the area under the ROC curve (AUC) and employed DeLong’s test for pairwise comparisons. Additionally, we constructed three nested association models:

Model A (Baseline): Incorporated traditional risk factors (hypertension, smoking, T2DM duration, HbA1c, age, total cholesterol, and serum creatinine).

Model B (Baseline + TyG): Enhanced the baseline model with the TyG index.

Model C (Baseline + TyHGB): Enhanced the baseline model with the TyHGB index.

The discrimination performance of these nested models was internally validated using the bootstrap method with 1000 re-samplings. Model discrimination capabilities were then compared using the AUC values derived from this validation.

Reclassification Improvement: We quantitatively assessed the incremental associative value of adding TyHGB to both the baseline model and the TyG-enhanced model using continuous net reclassification improvement (NRI) and integrated discrimination improvement (IDI).

## 3 Results

### 3.1 Study population characteristics

Our retrospective cross-sectional analysis comprised 1,382 adults diagnosed with type 2 diabetes mellitus. Within this population, 286 patients (20.7%) received a diagnosis of diabetic kidney disease. **Table 1** presents the baseline characteristics according to DKD status. The DKD group demonstrated significantly longer diabetes duration and elevated concentrations of multiple metabolic parameters compared to the non-DKD group. Specifically, we observed higher values for body mass index, fasting blood glucose, glycated hemoglobin, liver enzymes (ALT and AST), triglycerides, serum creatinine, uric acid, white blood cell count, and both TyG and TyHGB indices (all p<0.05). Conversely, high-density lipoprotein cholesterol levels were markedly reduced in DKD patients (p<0.05). Several parameters showed no significant intergroup differences, including age, cardiovascular disease or stroke prevalence, total bilirubin, total cholesterol, low-density lipoprotein cholesterol, blood urea nitrogen, and platelet count (all p > 0.05).

**Table 1:** Baseline characteristics of study participants.

Variable	Overall^1^	Non-DKD^1^	DKD^1^	P Value^2^
Sex				<0.001
0	538 (39%)	453 (41%)	85 (30%)	
1	843 (61%)	642 (59%)	201 (70%)	
Age (years)	57.29 (9.83)	57.16 (9.57)	57.79 (10.78)	0.6
Duration (years)	7.31 (5.89)	7.05 (5.66)	8.30 (6.59)	0.013
Hypertension				<0.001
0	723 (52%)	607 (55%)	116 (41%)	
1	658 (48%)	488 (45%)	170 (59%)	
Cardiovascular Disease				0.5
0	1,250 (91%)	994 (91%)	256 (90%)	
1	131 (9.5%)	101 (9.2%)	30 (10%)	
Stroke				0.4
0	1,303 (94%)	1,036 (95%)	267 (93%)	
1	78 (5.6%)	59 (5.4%)	19 (6.6%)	
Smoking				<0.001
0	957 (69%)	790 (72%)	167 (58%)	
1	424 (31%)	305 (28%)	119 (42%)	
Alcohol				0.008
0	989 (72%)	802 (73%)	187 (65%)	
1	391 (28%)	292 (27%)	99 (35%)	
Unknown	1	1	0	
BMI (kg/m²)	24.98 (3.24)	24.77 (3.16)	25.77 (3.44)	<0.001
FBG (mmol/L)	7.49 (2.24)	7.33 (2.10)	8.13 (2.63)	<0.001
HbA1c (%)	7.15 (1.43)	7.04 (1.37)	7.55 (1.59)	<0.001
TBIL (μmol/L)	13.08 (4.85)	13.15 (4.87)	12.82 (4.79)	0.3
ALT (U/L)	24.99 (15.19)	24.38 (14.47)	27.32 (17.51)	0.015
AST (U/L)	22.16 (8.95)	21.74 (7.82)	23.73 (12.25)	0.012
TG (mmol/L)	1.81 (1.74)	1.67 (1.46)	2.33 (2.46)	<0.001
TC (mmol/L)	4.77 (1.16)	4.74 (1.11)	4.92 (1.33)	0.2
HDL (mmol/L)	1.28 (0.39)	1.30 (0.40)	1.21 (0.32)	<0.001
LDL (mmol/L)	3.02 (0.97)	3.01 (0.97)	3.06 (0.97)	0.4
BUN (mmol/L)	6.01 (2.74)	5.91 (2.62)	6.36 (3.14)	0.2
SCr (μmol/L)	73.70 (37.38)	70.41 (18.86)	86.29 (72.11)	<0.001
UA (μmol/L)	349.41 (90.60)	343.54 (88.81)	371.85 (93.99)	<0.001
WBC (×10^9^/L)	6.39 (1.76)	6.26 (1.70)	6.90 (1.89)	<0.001
PLT (×10^9^/L)	230.66 (61.77)	230.59 (62.62)	230.94 (58.47)	0.7
TyG Index	9.04 (0.70)	8.96 (0.67)	9.32 (0.75)	<0.001
TyHGB Index	9.36 (2.65)	9.08 (2.43)	10.40 (3.15)	<0.001

^1^n (%); Mean (SD).

^2^Pearson's Chi-squared test; Wilcoxon rank sum test.

### 3.2 Multivariable analysis of TyHGB-DKD association

**Figure 1:**
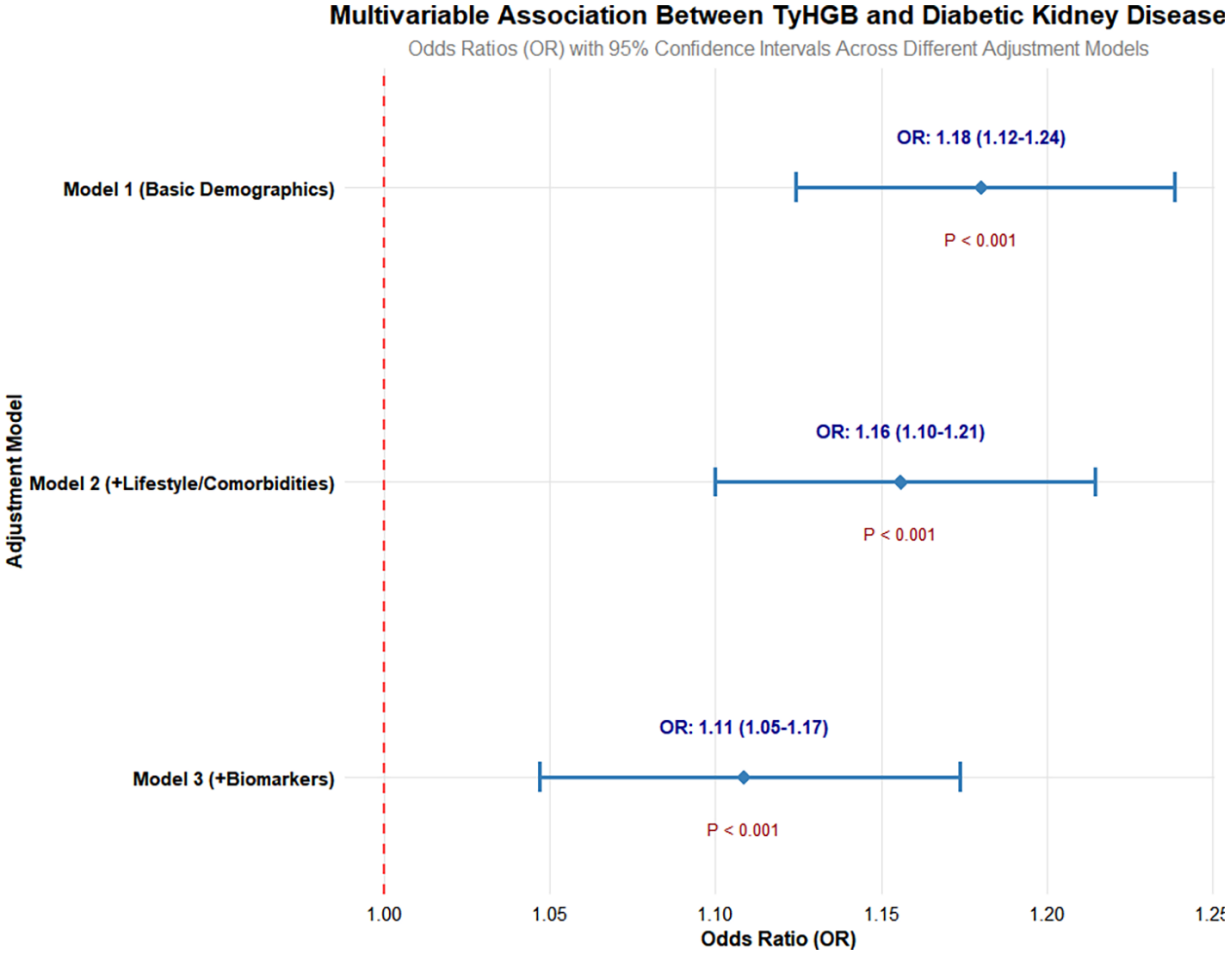
Multivariable adjusted associations between TyHGB index and diabetic kidney disease risk.

We employed multivariable logistic regression to examine the independent relationship between TyHGB and DKD (**Figure 1**). This association maintained statistical significance across progressively adjusted models:

Model 1 (demographic adjustment): OR = 1.18, 95% CI 1.12-1.24, p < 0.001.

Model 2 (lifestyle and comorbidity adjustment): OR = 1.16, 95% CI 1.10-1.21, p < 0.001.

Model 3 (primary fully adjusted model, excluding renal function markers): OR = 1.11, 95% CI 1.05-1.17, p < 0.001.

Sensitivity Analysis: When renal function markers (serum creatinine and blood urea nitrogen) were added to the fully adjusted model (Model 3a), the association between TyHGB and DKD remained virtually unchanged and highly significant (OR = 1.11, 95% CI: 1.05-1.17, p < 0.001), confirming the robustness of our primary finding.

### 3.3 Non-linear dose-response relationship

**Figure 2:**
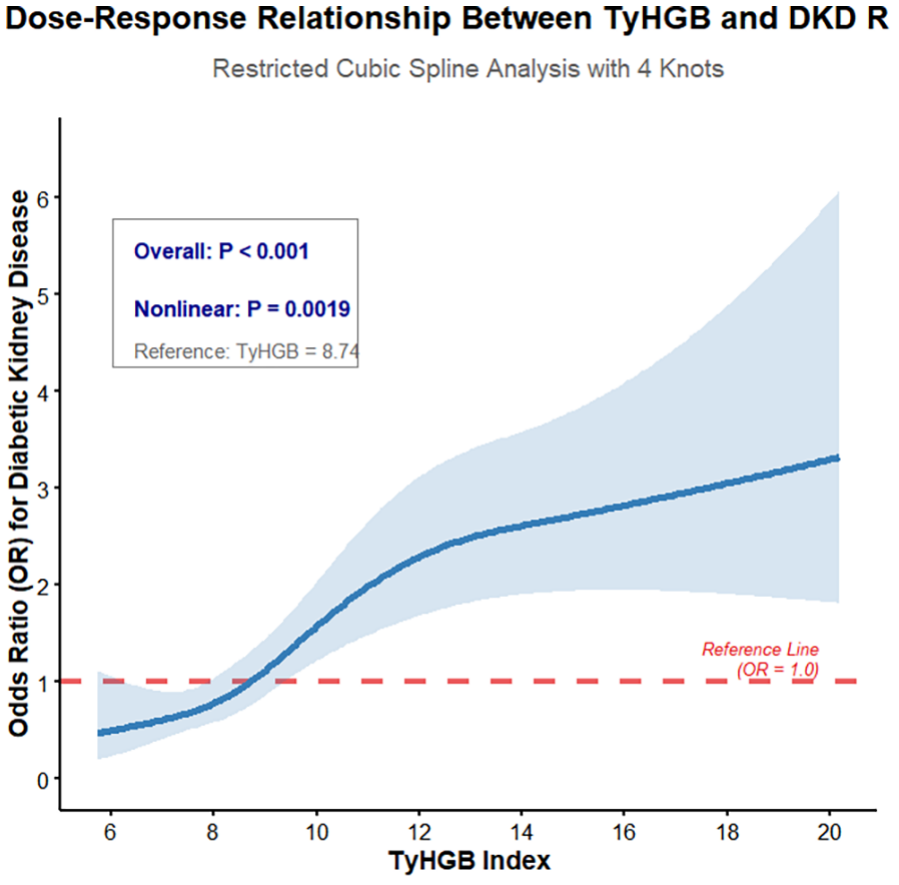
Non-linear relationship between TyHGB index and diabetic kidney disease risk: restricted cubic spline analysis.

Restricted cubic spline analysis identified a significant non-linear pattern in the TyHGB-DKD relationship (p = 0.002). As illustrated in **Figure 2**, DKD risk exhibited gradual increase until reaching a TyHGB threshold of approximately 8.74, beyond which risk escalation accelerated substantially with increasing TyHGB values. The overall association demonstrated high significance (p < 0.001), supporting a robust dose-response relationship. This non-linear configuration persisted following comprehensive adjustment for demographic, clinical, and biochemical covariates.

### 3.4 Subgroup analyses and effect modification

**Figure 3:**
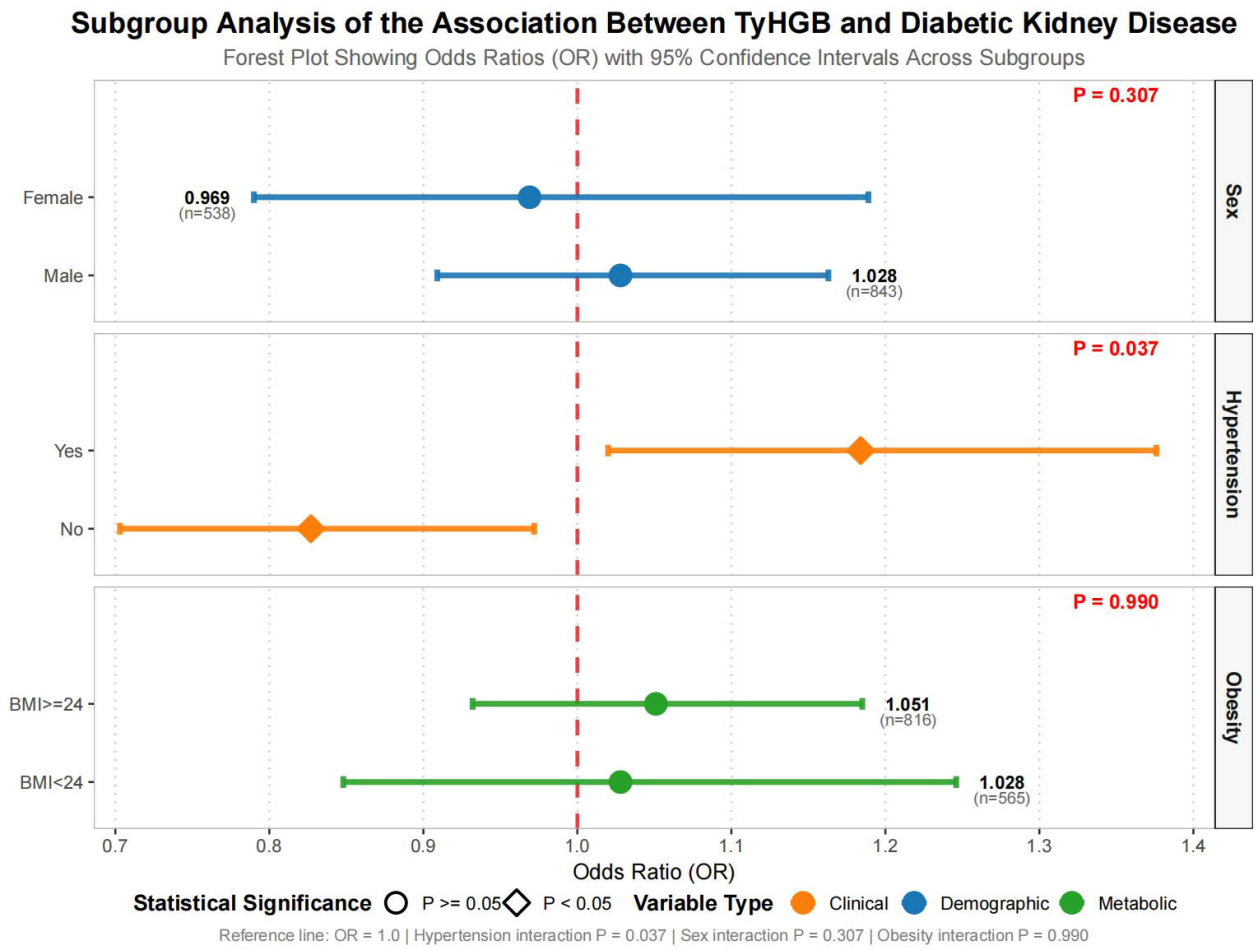
Subgroup analyses of the association between TyHGB index and diabetic kidney disease risk.

Stratified analyses evaluated the consistency of TyHGB-DKD associations across patient subgroups (**Figure 3**). Hypertension status significantly influenced this relationship (p for interaction = 0.037), with hypertensive patients showing stronger positive associations (OR = 1.213, 95% CI 1.040-1.415, p = 0.014) compared to normotensive individuals (OR = 0.835, 95% CI 0.702-0.992, p = 0.040). In contrast, neither sex (p = 0.307) nor obesity status (p = 0.990) demonstrated significant effect modification. The direction of association remained consistent across all subgroups, though statistical significance was not achieved in sex- or BMI-stratified analyses.

### 3.5 Associative performance assessment

**Figure 4:**
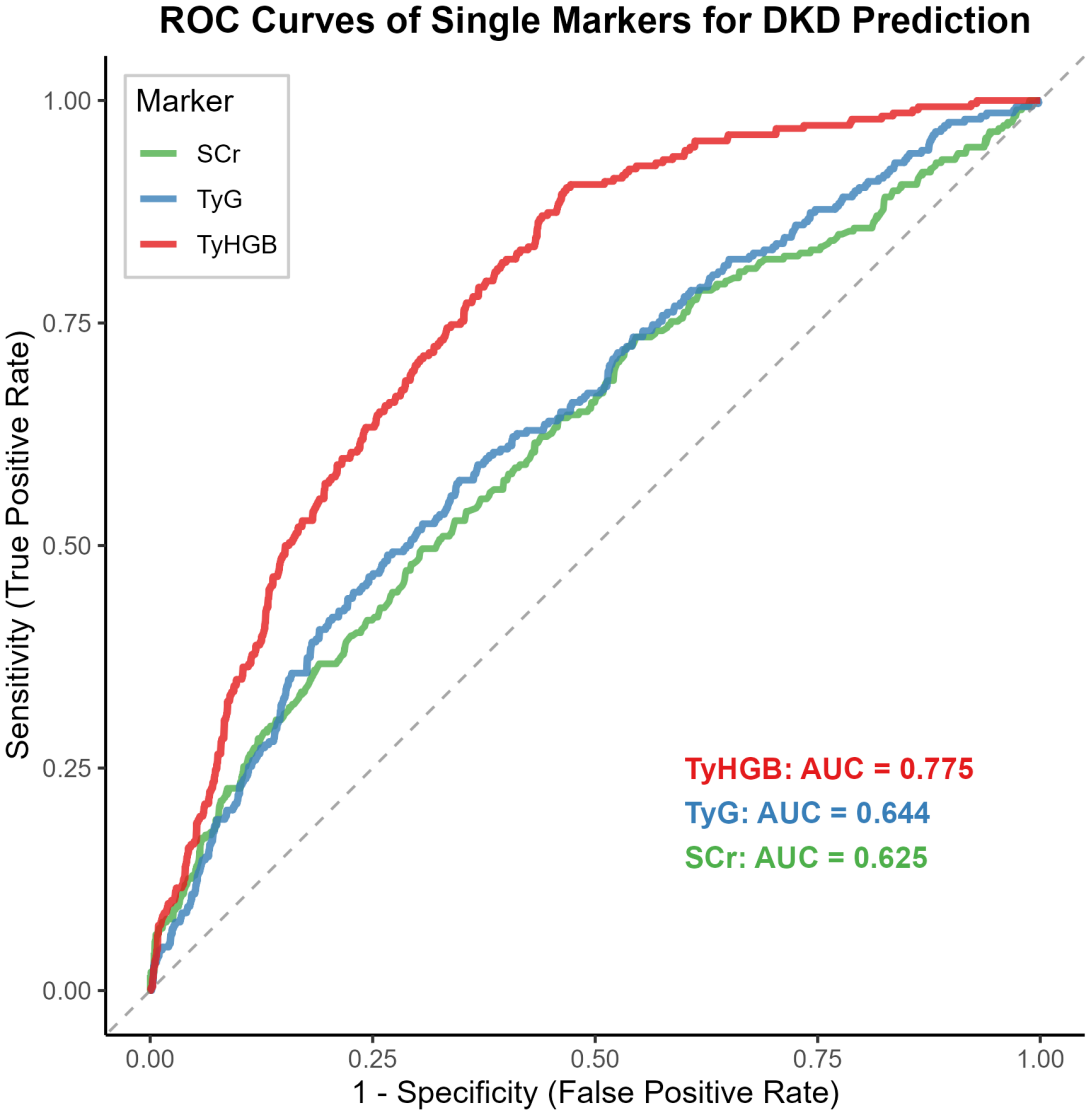
Receiver operating characteristic curves comparing TyHGB, TyG index, and serum creatinine for predicting diabetic kidney disease.

Receiver operating characteristic analysis evaluated the discriminative capacity of TyHGB against established biomarkers (**Figure 4**, **Table 2**). TyHGB achieved superior performance (AUC = 0.775, 95% CI: 0.747-0.803) compared to both the TyG index (AUC = 0.644, 95% CI: 0.608-0.681; p < 0.001) and serum creatinine (AUC = 0.625, 95% CI: 0.587-0.662; p < 0.001), establishing its advantage as a standalone marker.

**Table 2:** Comprehensive statistical analysis results.

Analysis type	Metric	Value	X95..CI...Components	P.value
Single Marker	TyHGB AUC	0.775	0.747-0.803	Reference
	TyG AUC	0.644	0.608-0.681	<0.001
	SCr AUC	0.625	0.587-0.662	<0.001
Nested Model	Model A AUC	0.715	0.682-0.749	Reference
	Model B AUC	0.728	0.696-0.761	0.060
	Model C AUC	0.788	0.759-0.817	<0.001
Improvement	NRI (Model C vs A)	0.647	Events: 0.252, Non-events: 0.395	N/A*
	IDI (Model C vs A)	0.067	ΔEvents: 0.053, ΔNon-events: -0.014	N/A*

*P-values for NRI and IDI require bootstrap estimation for statistical significance.

Model A: Hypertension + Smoking + Duration + HbA1c +Age + TC + SCr.

Model B: Model A + TyG, Model C: Model A + TyHGB.

### 3.6 Model enhancement and reclassification

**Figure 5:**
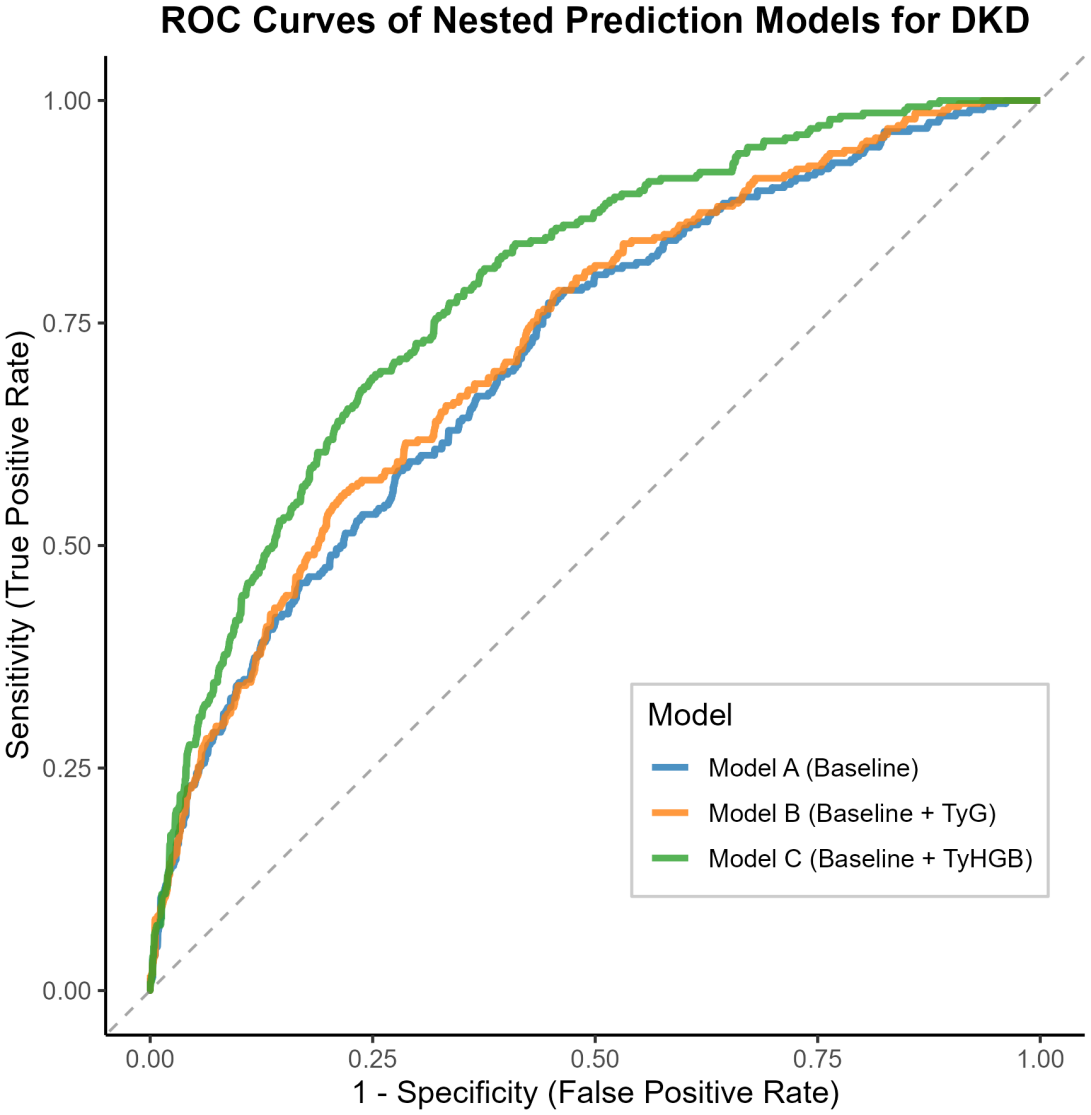
Predictive performance of nested models for diabetic kidney disease risk stratification.

Nested model construction evaluated TyHGB’s incremental associative value (**Figure 5**):

Baseline model: AUC = 0.715 (0.682-0.749).

Baseline + TyG: AUC = 0.728 (0.696-0.761).

Baseline + TyHGB: AUC = 0.788 (0.759-0.817).

TyHGB incorporation yielded significant improvement over both the baseline model (ΔAUC = 0.073, p < 0.001) and the TyG-enhanced model (ΔAUC = 0.060, p < 0.001). Furthermore, TyHGB addition produced substantial reclassification improvement (continuous NRI = 0.647) and enhanced integrated discrimination (IDI = 0.067), confirming its value for risk stratification.

## 4 Discussion

This community-based investigation establishes a robust and independent relationship between TyHGB—a novel composite metabolic index—and diabetic kidney disease prevalence in adults with type 2 diabetes. The association remained statistically significant following extensive adjustment for demographic, clinical, and biochemical variables. We identified a non-linear dose-response pattern, with DKD risk accelerating substantially beyond a TyHGB threshold of approximately 8.7. However, this threshold should be considered exploratory and requires validation in independent, prospective cohorts. As an independent marker, TyHGB demonstrated enhanced discriminative ability for DKD compared to both the TyG index and serum creatinine, achieving an AUC of 0.775. Incorporation of TyHGB into conventional risk association models significantly improved both discrimination and reclassification accuracy. Collectively, these findings position TyHGB as a practical, integrative metabolic indicator that delivers meaningful incremental prognostic value for DKD risk assessment.

The complex pathogenesis of DKD involves multiple interconnected mechanisms, with insulin resistance, dyslipidemia, chronic inflammation, and oxidative stress representing key pathophysiological contributors (22, 23). The triglyceride-glucose index has emerged as a well-validated IR surrogate that correlates with DKD risk across multiple epidemiological studies (24, 25). Complementary evidence from Jiang et al. confirmed that both TyG and TyG-BMI independently associate with DKD in newly diagnosed type 2 diabetes patients, though with limited diagnostic accuracy (AUC = 0.57) (26). Supporting these observations, Li et al. documented positive correlations between TyG index values and albuminuria severity (27). However, the TyG index predominantly reflects glucose and triglyceride metabolism, potentially overlooking other critical pathways involving anti-inflammatory lipid regulation and adiposity-related mechanisms.

Our results reinforce the conceptual framework that composite indices capturing broader metabolic dysregulation may offer superior associative capability. TyHGB advances beyond conventional TyG metrics by incorporating the TG/HDL-C ratio—an established marker of atherogenic dyslipidemia and IR—together with fasting glucose and BMI measurements. This integrated approach enables TyHGB to represent the complex interplay among dyslipidemia, glucotoxicity, and adiposity. The inclusion of HDL-C is particularly relevant given its documented anti-inflammatory, antioxidant, and endothelial-protective functions, with low levels independently associated with renal functional decline (28, 29). Similarly, BMI serves as an indicator for obesity-mediated renal injury, which operates through glomerular hyperfiltration, leptin resistance, and chronic inflammatory pathways (30). Thus, TyHGB provides a more comprehensive assessment of the multifaceted metabolic disturbances driving DKD progression.

Our restricted cubic spline analysis identified a significant non-linear relationship between TyHGB values and DKD risk. The observed threshold effect at TyHGB = 8.74 suggests a critical transition point in metabolic homeostasis, beyond which renal injury risk increases substantially. This pattern aligns with established concepts of threshold effects in microvascular complication pathogenesis. Previous investigations have documented similar non-linear associations for other metabolic indices, including the U-shaped relationship between TyG index and diabetic retinopathy reported by Zhou et al. (31). Our findings extend this paradigm to the TyHGB-DKD relationship, emphasizing the importance of evaluating non-linearity in metabolic risk assessment.

Stratified analyses provided clinically important insights regarding effect modification. The strengthened TyHGB-DKD association in hypertensive individuals implies that metabolic dysregulation and hemodynamic stress may synergistically accelerate renal damage through multiple complementary pathways in the context of hypertension, thereby enhancing the TyHGB-DKD relationship. Chronic low-grade inflammation and upregulation of pro-inflammatory cytokines (e.g., IL-6, TNF-α) in the comorbidity of hypertension and insulin resistance amplify the glomerular and interstitial inflammatory injury triggered by dysglycemia, dyslipidemia, and obesity (32). Concurrently, oxidative stress and lipid peroxidation can damage the glomerular basement membrane and podocytes, promoting proteinuria and the deterioration of filtration function (33). Furthermore, sympathetic nervous system overactivation and hemodynamic alterations (glomerular hypertension, hyperfiltration) exacerbate these metabolically induced pathological processes through sustained mechanical stress and microcirculatory disturbances (34). These interactions collectively manifest in shared pathways such as RAAS activation, mitochondrial dysfunction, and endothelial impairment, forming a pathogenic network that drives DKD progression. Conversely, the consistent associations observed across sex and BMI categories reinforce TyHGB’s generalizability as a risk marker, demonstrating its utility beyond specific demographic or adiposity subgroups.

TyHGB’s superior associative performance (AUC = 0.775) relative to TyG index (AUC = 0.644) and serum creatinine (AUC = 0.625) underscores its clinical potential. These observations are consistent with emerging research evaluating TyHGB in other contexts, including its excellent associative accuracy for gestational diabetes mellitus (AUC 0.707-0.859) as demonstrated by Xu et al. (17). The conceptual approach of enhancing TyG through additional parameters follows established methodology, exemplified by Er and Li et al.’s development of TyG-BMI as a superior IR marker (35, 36); our work successfully translates and validates this approach specifically for DKD risk assessment.

The incorporation of TyHGB into baseline clinical models produced significant improvement in discriminant capacity (AUC increase from 0.715 to 0.788) and reclassification accuracy (NRI = 0.647; IDI = 0.067). These findings indicate that TyHGB captures unique risk information not fully represented by conventional factors, supporting its potential integration into clinical risk scores and electronic health record-based screening protocols.

Furthermore, our findings proved robust in a sensitivity analysis that included renal function markers in the adjustment model, mitigating concerns about over-adjustment bias. While our study benefits from real-world community data, comprehensive confounder adjustment, and sophisticated non-linearity testing that identified an actionable clinical threshold, several limitations merit consideration. The retrospective single-center design may affect generalizability, though multi-site data sourcing enhances real-world applicability. Potential unmeasured confounders, including dietary patterns, physical activity levels, and specific medications (e.g., SGLT2 inhibitors, GLP-1 receptor agonists), could influence observed relationships. Additionally, DKD diagnosis relied on UACR and eGFR criteria without biopsy confirmation, though this approach aligns with current clinical practice guidelines (37). Future multi-center, prospective, and multi-ethnic studies, with serial TyHGB measurements, are needed to validate our findings and elucidate the causal mechanisms.

## 5 Conclusion

The triglyceride high-density cholesterol-glucose body index (TyHGB)—a readily calculable composite of TG/HDL-C ratio, fasting glucose, and BMI—demonstrates independent non-linear association with diabetic kidney disease in type 2 diabetes patients. It significantly enhances risk discrimination beyond conventional factors and established indices. TyHGB’s practical derivation and robust performance support its potential role in DKD screening and stratification protocols, warranting additional prospective validation and exploration within integrated assessment frameworks.

## Data Availability

The datasets presented in this article are not readily available because they constitute electronic health records sourced from a network of community health centers, containing sensitive patient information. The data were used under license for the current retrospective study in a de-identified format, in compliance with the ethical approvals and data access agreements granted by the Medical Ethics Committee of Longhua District People’s Hospital of Shenzhen and the Data Governance Committee of the Eighth Affiliated Hospital of Sun Yat-sen University (Futian). Requests to access these datasets should be directed to the corresponding author and will require a formal data sharing agreement and subsequent permission from the aforementioned ethics and data governance committees. Requests to access these datasets should be directed to the corresponding author, Email: 54574963@qq.com.
